# Targeted suppression of heme oxygenase-1 by small interference RNAs inhibits the production of bilirubin in neonatal rat with hyperbilirubinemia

**DOI:** 10.1186/1471-2199-10-77

**Published:** 2009-08-01

**Authors:** Jinyong Wu, Wen Su, Youxin Jin, Yi Shi, Chune Li, Wenwei Zhong, Xuehong Zhang, Zili Zhang, Zhenwei Xia

**Affiliations:** 1 Ruijin Hospital Affiliated to School of Medicine, Shanghai Jiao Tong University, Shanghai, PR China; 2 School of Life Science and Technology, Shanghai Jiao Tong University, Shanghai, PR China; 3 State Key Laboratory of Molecular Biology, Institute of Biochemistry and Cell Biology, Shanghai Institutes for Biological Sciences, Chinese Academy of Sciences, Shanghai, PR China; 4 Department of Pediatrics, Oregon Health and Sciences University, Portland, Oregon, USA

## Abstract

**Background:**

Excessive accumulation of bilirubin contributes to neonatal hyperbilirubinemia in rats. Heme oxygenase (HO) is one of the rate-limiting enzymes in catabolizing heme to bilirubin. In the present study, we investigated whether suppression of rat HO-1 (rHO-1) expression by small interference RNAs (siRNAs) reduces bilirubin levels in hyperbilirubinemic rats.

**Results:**

Four pairs of siRNA targeting rHO-1 mRNA were introduced into BRL cells and compared for their inhibitory effect on the expression of *rHO-1 *gene and production of rHO-1 protein. The siRNA exhibiting the most potent effect on HO-1 expression and activity was then administered intraperitoneally to 7 to 9-day-old rats with hyperbilirubinemia. The siRNA distributed mostly in the liver and spleen of neonatal rat. Serum bilirubin levels and hepatic HO-1 expression were further evaluated. Systemic treatment of siRNA targeting rHO-1 reduced hepatic HO-1 expression and decreased the serum bilirubin levels in a time- and dose-dependent manner, and siRNA decreased the indirect bilirubin levels more effectively than Sn-protoporphyrin (SnPP), an HO-1 inhibitor.

**Conclusion:**

siRNA targeting rHO-l attenuates hepatic HO-1 expression and serum bilirubin levels. Thus this study provides a novel therapeutic rationale for the prevention and treatment of neonatal hyperbilirubinemia.

## Background

Neonatal hyperbilirubinemia is a common medical condition in newborn mammalian. About 60% term infants and 80% preterm infants develop hyperbilirubinemia within one week after birth. Neonatal hyperbilirubinemia is mainly due to the accumulation of bilirubin as a result of metabolic disturbance caused by various factors. Normally, serum bilirubin levels increase within 3 to 5 days after birth, and then begin to decline [1]. However, under pathologic states, serum bilirubin, especially indirect bilirubin, can reach an exceedingly high level which leads to neurotoxicity, namely kernicterus, and eventually results in permanent neurological damage or even death [2].

Bilirubin is produced by the degradation of heme. Heme oxygenase (HO) is the initial and rate-limiting enzyme in this catabolic process. HO consists of three isozymes: HO-1, HO-2 and HO-3. Heme released from degraded fetal red blood is a potent inducer of HO-1 in the neonatal period, which is responsible for the increase of serum bilirubin levels and the development of hyperbilirubinemia. Inhibition of HO-1 expression or its enzymatic activity concurs to the reduction of bilirubin levels [3]. Therefore, it is plausible to target HO-1 as a novel therapeutic rationale for the treatment of neonatal hyperbilirubinemia. In this study, we aimed to assess the efficacy of direct inhibition of bilirubin production by suppressing HO-1 in a neonatal rat hyperbilirubinemia model.

Small interference RNA (siRNA) technology utilizes short double-stranded RNA to specifically inhibit the transcription of a given target gene [4]. This technique has been proven to be a promising method in treating a number of diseases, such as hereditary diseases, viral hepatitis, and certain cancers [5]. Furthermore, RNA interference as a means of treating age-related macular degeneration has entered into a clinical trial [6]. These advances provide a theoretical and experimental basis to treat neonatal hyperbilirubinemia using siRNA. Previously, we demonstrated that siRNA specifically inhibits the expression of human HO-1 (hHO-1) in human liver cell line HL-7702 [7]. In this paper, methoxyl siRNA targeting rat HO-1 (rHO-1) mRNA was used to suppress HO-1 in neonatal hyperbilirubinemia rat model. This study tested the feasibility of preventing and treating neonatal hyperbilirubinemia and bilirubin toxic encephalopathy by targeting *HO-1 *gene.

## Results

### siRNA Transfection Efficiency

**Figure 1:**
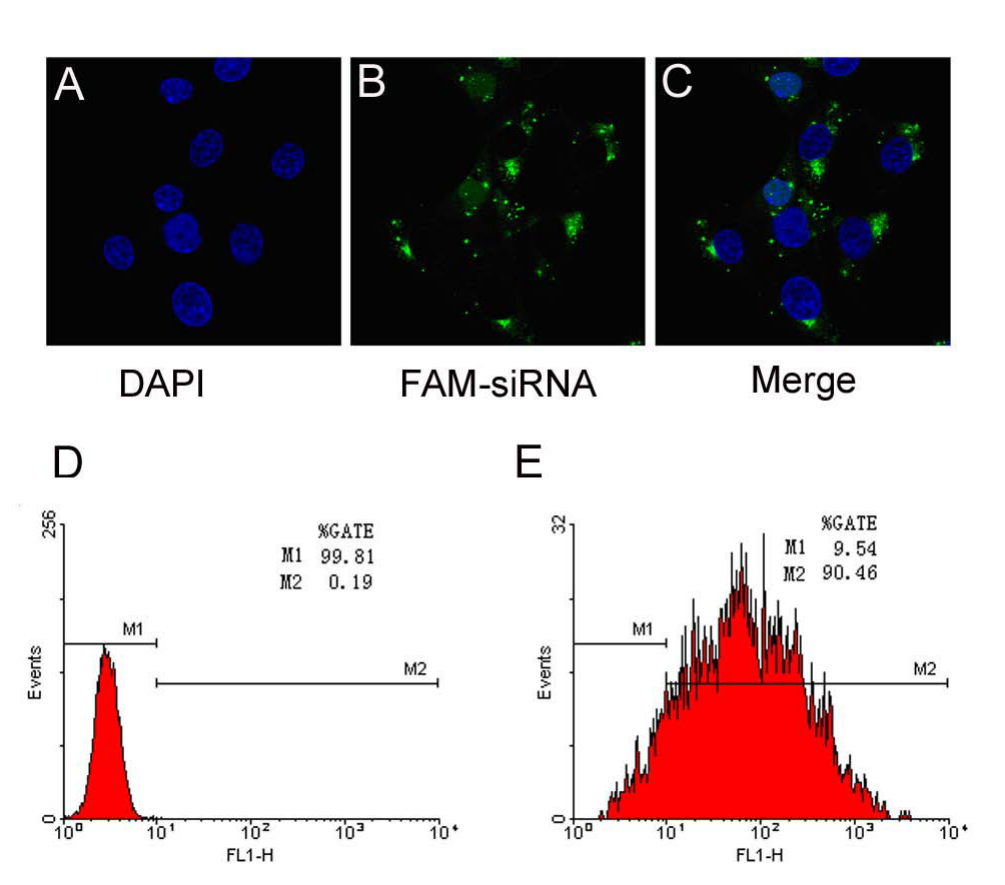
**Detection of siRNA transfection efficiency**. Fifty nmol/L FAM labeled NC-siRNA was transfected into the cells for 6 hours. The nuclei were stained with DAPI. Confocal microscopy (×60) and flow cytometry were employed to detect the transfection efficiency (upper figure A, B and C), siRNA entered the cells, and primarily distributed in the cytoplasm. The lower figure was the result from flow cytometric analysis, D contained untransfected cells, E contained FAM-siRNA transfected cells. The results were analyzed with Win MDI29 software, which indicated that the siRNA transfection efficiency in BRL cells was approximately 90%.

To determine the transfection efficiency of siRNA, a carboxy-fluorescein (FAM) labeled negative control (NC)-siRNA was transfected into BRL cells, and the nuclei were stained with 4',6-diamidino-2-phenylindole (DAPI) after 6 hours. Confocal microscopy showed that 6 hours post-transfection, siRNA entered the cells and primarily distributed in the cytoplasm (Figure 1A to 1C). Furthermore, flow cytometry demonstrated the siRNA transfection efficiency in BRL cells was up to 90% (Figure 1D and 1E).

### Down-Regulation of *rHO-1 *Gene Expression by siRNA

**Figure 2:**
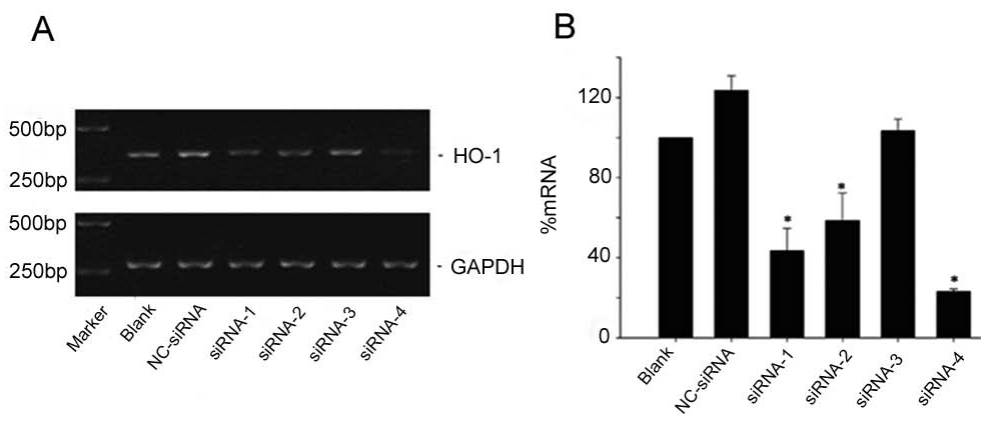
**The inhibitory effect of four pairs of siRNAs on HO-1 mRNA**. Four pairs of double-strand siRNAs were transfected into BRL cell for 24 hours. rHO-1 mRNA levels were analyzed by RT-PCR and were found to be reduced by more than 75% by siRNA-4, 57% by siRNA-1, and 42% by siRNA-2. siRNA-3 did not exhibit any significant inhibitory effect. A: Results of RT-PCR. B. Statistical analysis. Data represent the results from three independent experiments. *P < 0.01, compared with NC-siRNA group.

Quantitative gel gray scale scanning was used to compare the inhibitory effect of four pairs of siRNA targeting the *rHO-1 *gene. siRNA-4 showed the most potent inhibition on rHO-1 mRNA expression (greater than 75%, Figure 2). Therefore, siRNA-4 was selected for the rest of the experiments.

### Dose- and Time-Dependent Down-Regulation of rHO-1 by siRNA

**Figure 3:**
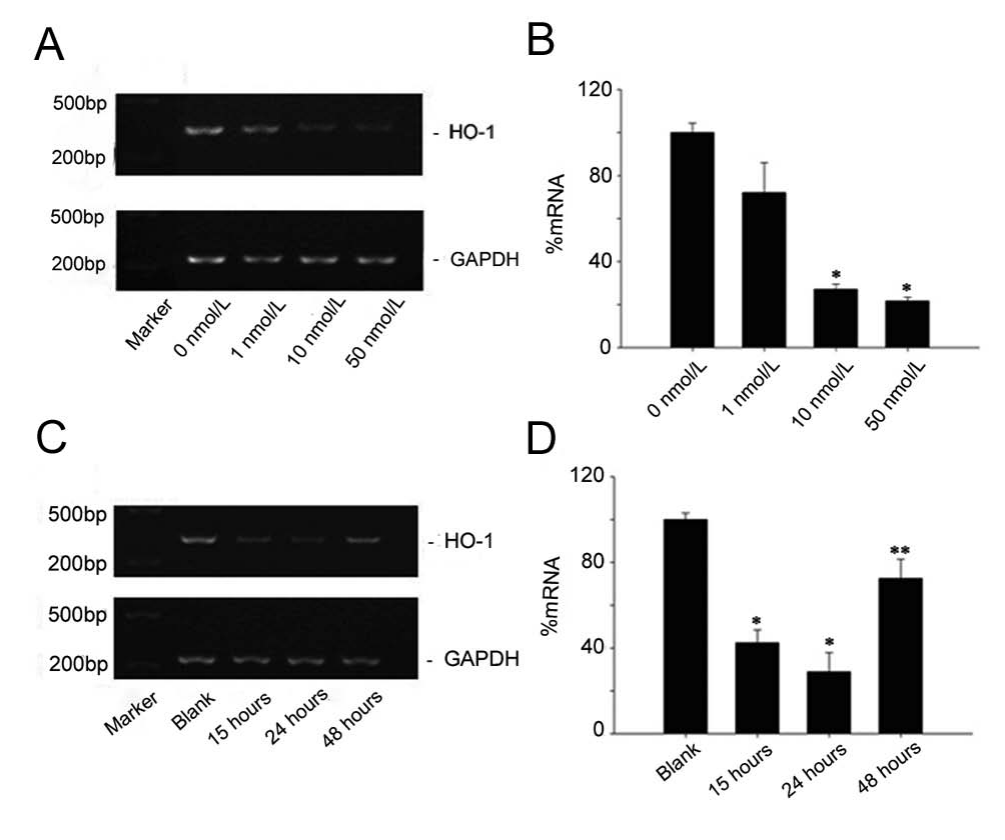
**Dose- and time-dependent down-regulation of rHO-1 by siRNA**. A and B. The dose-dependent inhibitory effect of siRNA (*P < 0.01, compared with untransfected controls). The doses of siRNA used in the experiments were 0 nmol/L, 1 nmol/L, 10 nmol/L and 50 nmol/L. C and D. Time-dependent inhibitory effect of siRNA. The time points were 15 hours, 24 hours and 48 hours at a siRNA dose of 10 nmol/L. The maximum inhibitory effect of siRNA-4 was observed at 24 hours. (*P < 0.01 or **P < 0.05). The results represent values derived from three independent experiments.

BRL cells were transfected with 1, 10 and 50 nmol/L siRNA-4, respectively, and collected at 24 hours post-transfection. RT-PCR was performed to determine the HO-1 mRNA level. The expression of rHO-1 in the BRL cells transfected with 1, 10 and 50 nmol/L siRNA were inhibited approximately 30%, 72% and 80%, respectively. Thus, the inhibitory effect of siRNA-4 on rHO-1 exhibited a dose-dependent relationship (Figure 3A and 3B).

Ten nmol/L siRNA-4 was transfected into BRL cells, and the rHO-1 expression was examined at 15, 24 and 48 hours post-transfection as demonstrated in Figure 3C and 3D. The maximum inhibitory effect of siRNA-4 was observed at 24 hours.

### Effect of Hemin and siRNA on rHO-1 Protein

**Figure 4:**
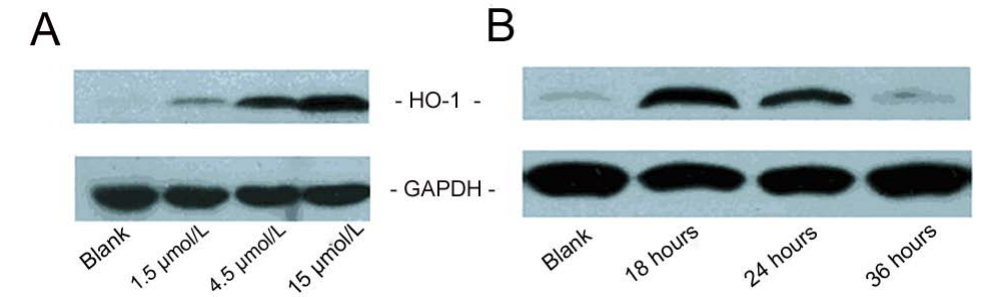
**Effect of hemin on rHO-1 protein**. A. The effect of hemin at different concentrations on the level of HO-1 protein. BRL cells were treated with 1.5 μmol/L, 4.5 μmol/L and 15 μmol/L hemin to induce the HO-1 expression, result of Western Blot 18 hours after the treatment indicated that the level of HO-1 protein was in a hemin dose-dependent way to increase. B. The change of HO-1 protein level induced by 4.5 μmol/L hemin at different time points. Eighteen hours after treatment with hemin, the level of HO-1 protein reached its peak.

Hemin, an HO-1 inducer, was selected to induce the expression of rHO-1. BRL cells were incubated with 1.5, 4.5 and 15 μmol/L hemin, and collected 18 hours post-induction. The level of rHO-1 protein was determined by Western Blot analysis. Hemin induced rHO-1 in a dose-dependent manner. However, cell toxicity was observed at the higher concentrations of hemin. Thus, 4.5 μmol/L was selected as the working concentration for rHO-1 induction (Figure 4A). Cells treated with 4.5 μmol/L hemin for 18, 24 and 36 hours were collected to determine the level of rHO-1 protein by Western Blot analysis. Eighteen hours after induction, rHO-1 level peaked and then declined over time. Therefore, the optimized induction time of rHO-1 by hemin was determined to be18 hours (Figure 4B).

**Figure 5:**
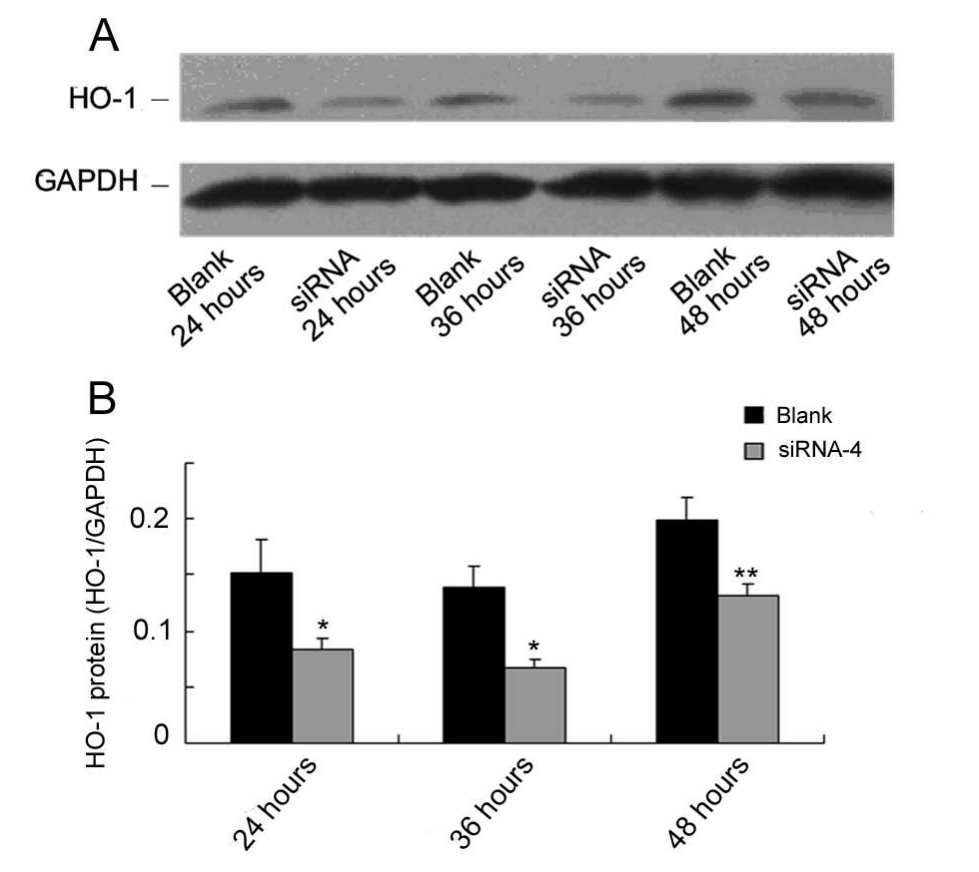
**Effect of siRNA-4 on hemin-induced HO-1 protein level**. BRL cells were transfected with 10 nmol/L siRNA-4 and collected at 24, 36 and 48 hours respectively after the transfection. Eighteen hours before the collection, 4.5 μmol/L hemin was added into all the groups to induce HO-1 expression. Compared to the blank group, the inhibitory rates of siRNA to HO-1 at 24, 36 and 48 hours after transfection were 53%, 64% and less than 30%, respectively. * *P *< 0.01 or ** *P *< 0.05 A. Results of Western Blot. B. Statistical results. Data represent the results from three independent experiments.

The inhibitory effect of siRNA-4 on rHO-1 is shown in Figure 5A and 5B. Compared with the negative control, siRNA inhibited the hemin-induced expression of HO-1 in a time-dependent manner. The inhibitory effect of siRNA reached the maximum level of 64% at 36 hours post-transfection.

### Effect of siRNA-4 on Enzymatic Activity of rHO-1

In order to verify our assumption that the suppression of HO-1 will ultimately lead to a reduction of bilirubin *in vitro*, homogenates from BRL cells transfected with and without siRNA-4 were used to measure bilirubin production by the rHO-1 enzymatic assay. The activity of rHO-1 treated with hemin and siRNA-4 was 3.33 ± 0.17 and 2.31 ± 0.17 nmol bilirubin mg protein^-1 ^h^-1^, respectively (Table 1).

**Table 1:** The Enzymatic Activity of rHO-1 After Hemin Induction and siRNA-4 Transfection

	Control (*n *= 4)	Hemin (*n *= 4)	Hemin + siRNA-4 (*n *= 4)
Enzymatic activity (Units)	1.15 ± 0.07	1.35 ± 0.07	0.95 ± 0.06
Specific activity (nmol bilirubin mg protein^-1 ^h^-1^)	2.62 ± 0.16	3.33 ± 0.17*	2.31 ± 0.17

* *P *< 0.05, compared with control group

### Distribution of FAM-siRNA in Neonatal Rat

**Figure 6:**
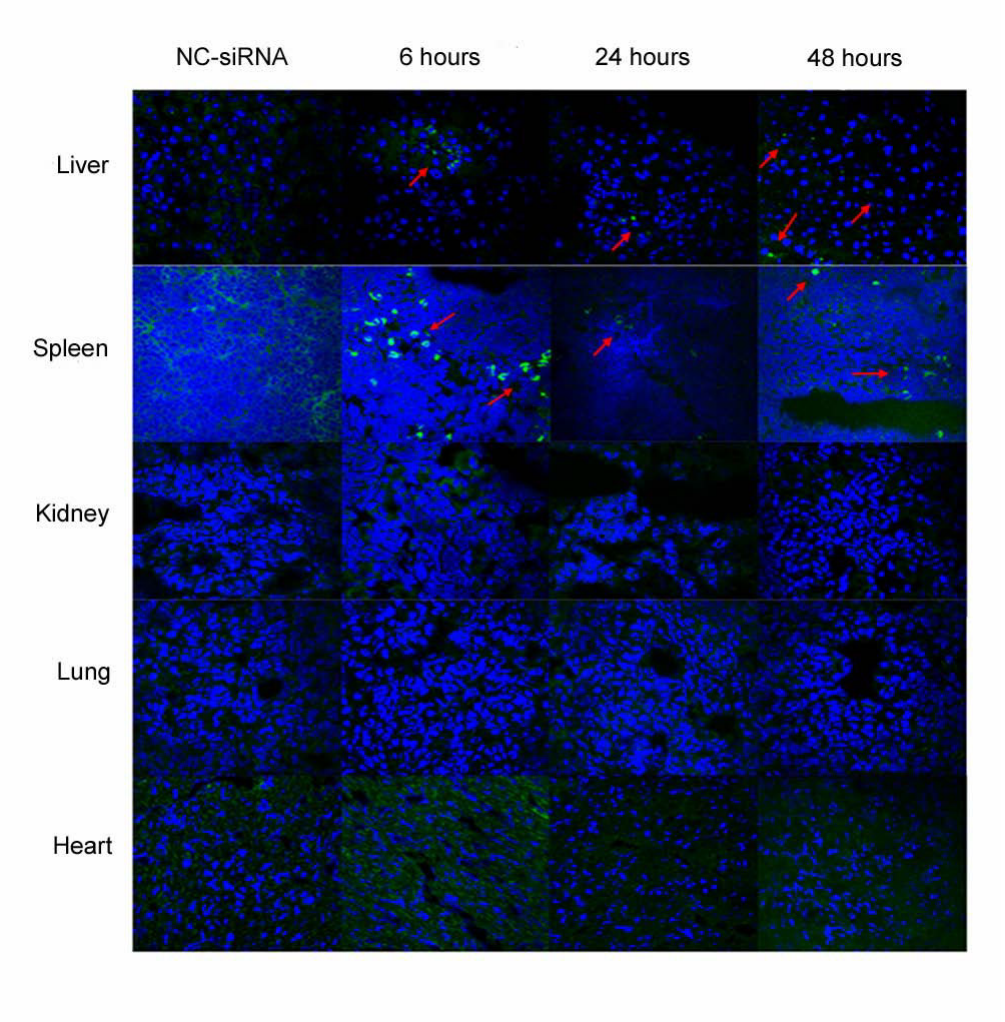
**The distribution of FAM-siRNA-4 in neonate rat**. Seven-day old rats were injected intraperitoneally with FAM labeled siRNA-4. At 6, 24 and 48 hours after injection, the distribution of FAM-siRNA was observed in liver, spleen, kidney, heart and lung (×60). The results localized siRNA-4 mainly to liver and spleen. Data represent the results from one of three independent experiments.

To confirm the targeting effect of siRNA in vivo, FAM labeled siRNA-4 was injected intraperitoneally into 7-day-old neonatal Sprague-Dawley (SD) rats. Confocal microscopy showed the distribution of fluorescence in the tissues at 6, 24 and 48 hours after injection, and the siRNA distributed predominantly in the liver and spleen (Figure 6).

### Dose- and Time-Dependent Reduction of the Serum Bilirubin and rHO-1 Levels by siRNA in a Neonatal Hyperbilirubinemia Rat Model

**Figure 7:**
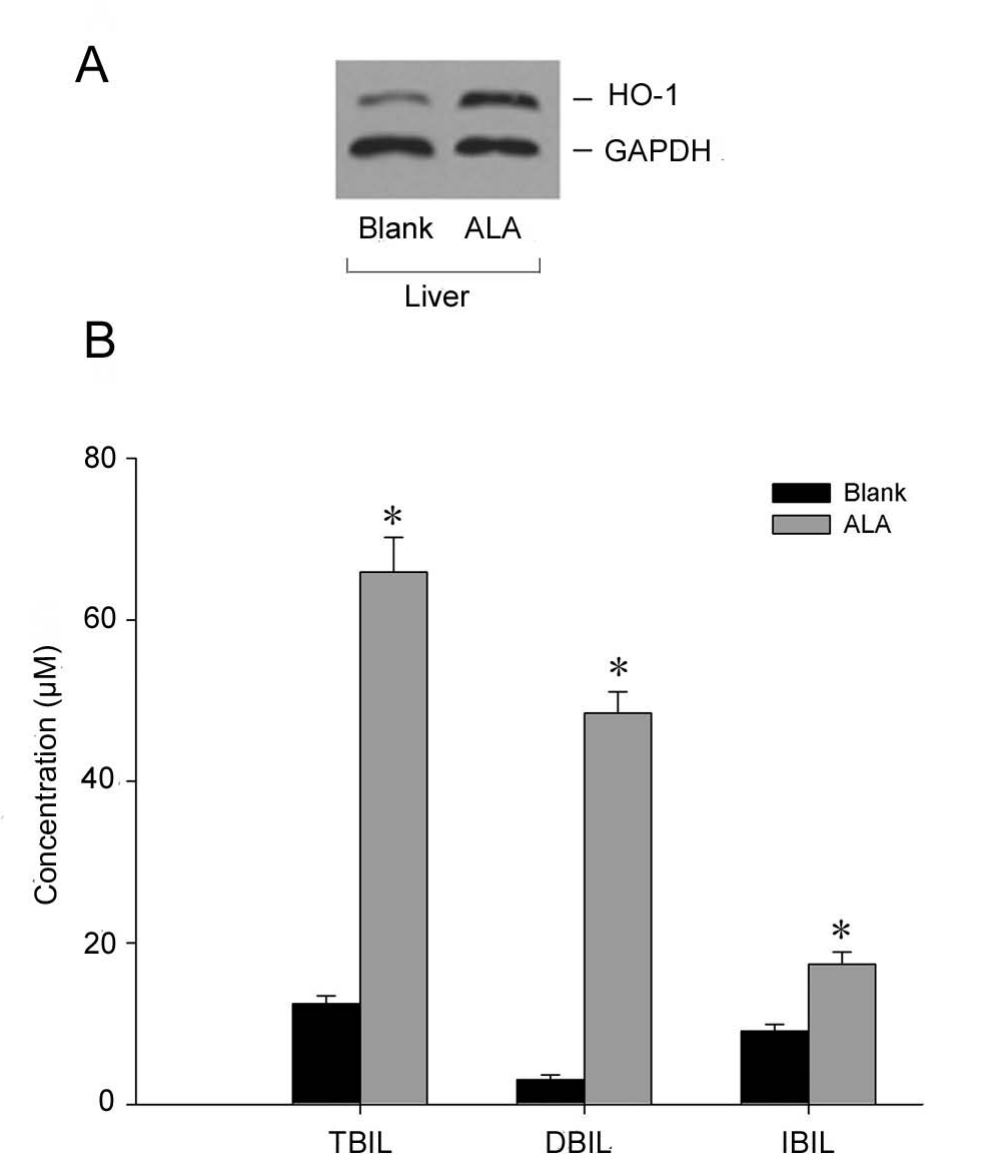
**The level of HO-1 protein in the liver of rats is significantly enhanced by ALA treatment**. A. Protein level of HO-1 in the liver, B. Concentration of serum bilirubin, TBIL: total bilirubin; DBIL: direct bilirubin, IBIL: indirect bilirubin. Data represent the results from one of three independent experiments. Each group contained four mice.

In light of the *in vitro *results, we sought to determine if siRNA inhibited serum bilirubin *in vivo*. Seven to 9-day-old SD rats were administered intraperitoneally with δ-aminolevulinic acid (ALA) 50 μmol/100 g body weight (bw) to induce hyperbilirubinemia. Western Blot analysis showed that the level of HO-1 significantly increased in the liver of these rats (Figure 7A). This is consistent with recent reports in the literature [3]. In addition, the levels of serum total bilirubin, direct bilirubin and indirect bilirubin all increased (Figure 7B). This suggests that we have successfully established a rat model of neonatal hyperbilirubinemia.

**Figure 8:**
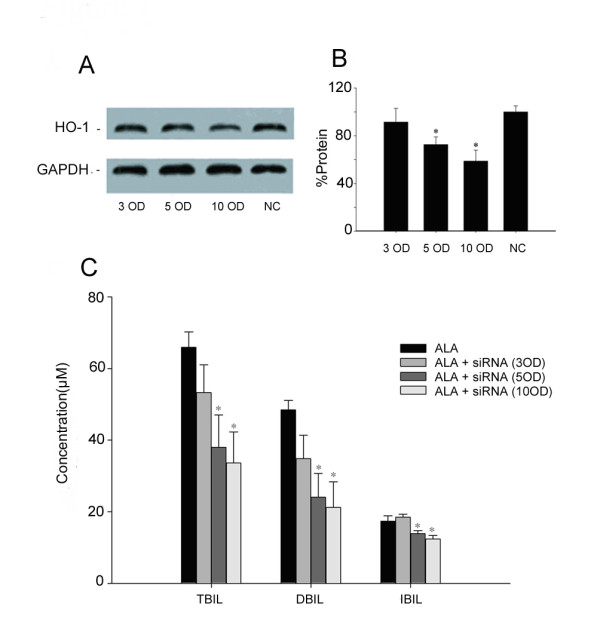
**Dosage-dependent reduction in the levels of serum bilirubin and rHO-1 by siRNA-4 in neonatal hyperbilirubinemia rats models**. A and B: The inhibitory effect of siRNA-4 on rHO-1 in liver increased concomitantly with increased injection doses. The inhibitory rates at 10 OD/20 g bw, 5 OD/20 g bw and 3 OD/20 g bw dosage were 42%, 30% and 10%, respectively. C. Effect of different dosages of siRNA-4 on the levels of bilirubin. *P < 0.05, compared with control group. Data represent the results from one of three independent experiments. Each group contained 4–6 mice.

**Figure 9:**
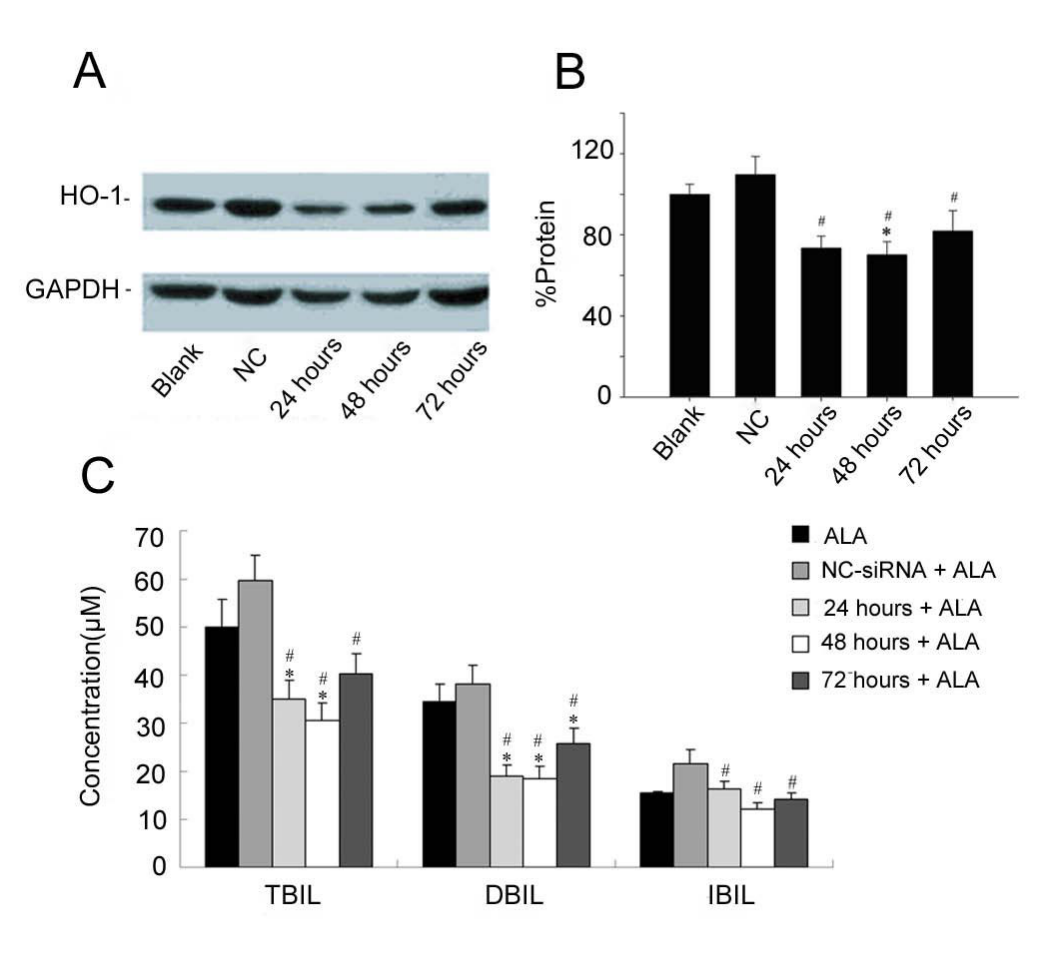
**Time-dependent reduction in the levels of serum bilirubin and rHO-1 by siRNA in neonatal hyperbilirubinemia rats models**. A and B: The inhibitory rate of siRNA-4 on HO-1 protein 48 hours after injection was 33%, after 72 hours, it was in a time-dependent decrease. No inhibitory effect of siRNA on HO-1 protein was found in NC group. C. Effect of 10 OD/20 g bw siRNA-4 on levels of bilirubin at different time points. *P < 0.05, compared with blank group; #*P *< 0.05, compared with NC group. Data represent the results from one of three independent experiments. Each group contained 4–6 mice.

Western Blot analysis revealed that *in vivo *siRNA-4 treatment reduced rHO-1 product in the liver in a concentration-dependent manner (Figure 8A and 8B). The levels of total, direct and indirect bilirubin decreased considerably after siRNA-4 injection at doses of 5 OD/20 g and 10 OD/20 g bw (Figure 8C). This effect was concentration-dependent. However, the decrease of total, direct and indirect bilirubin was not significant at 3 OD/20 g bw (Figure 8C). In rats treated intraperitoneally with 10 OD/20 g bw of siRNA-4 for three times, no abnormal behavior was observed. The changes of both serum bilirubin and rHO-1 indicate that the effective treatment time of siRNA-4 was 48 hours because the inhibitory effect decreased at 72 hours. The control group showed no significant change in either bilirubin levels or rHO-1 in the liver, suggesting the inhibitory effect of siRNA-4 is HO-1 specific (Figure 9A to 9C).

### Effect of siRNA and SnPP in a Neonatal Hyperbilirubinemia Rat Model

**Figure 10:**
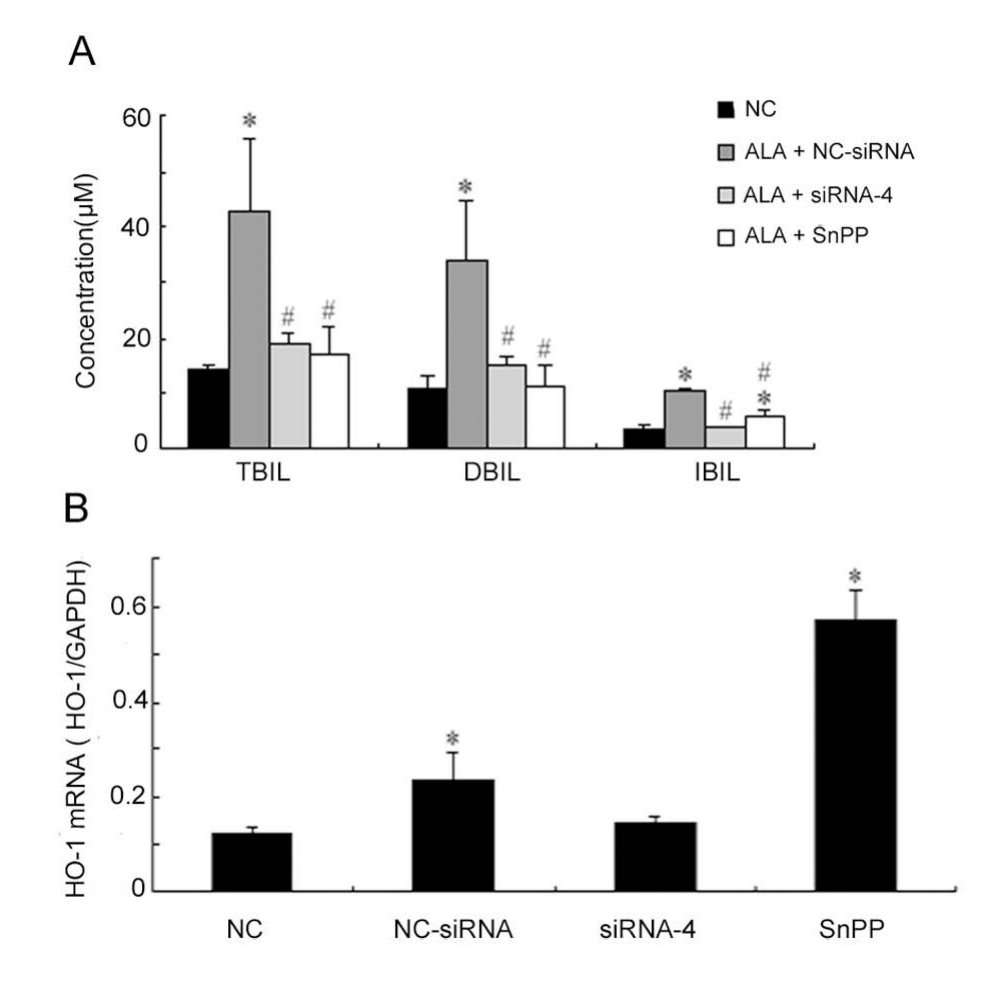
**Inhibiting effect of siRNA and SnPP on HO-1 in neonatal hyperbilirubinemia rats modes**. A. siRNA-4 and SnPP were injected intraperitoneally into 7-day-old rats respectively, and serum total bilirubin levels decreased significantly in both group. Compared with SnPP, siRNA-4 was more effective at reducing indirect bilirubin. * *P *< 0.05, compared with NC group; # *P *< 0.05, compared with ALA+NC-siRNA group. B. The expression of rHO-1 in liver was analyzed by Real-Time PCR. The level of HO-1 expression in siRNA-4 treatment group was significantly lower than that in NC-siRNA group. However, the HO-1 level increased significantly in SnPP group. * *P *< 0.05 compared with NC group. Data represent the results from one of two independent experiments. Each group contained 6–8 mice.

We next compared the suppressive effect of siRNA and Sn-protoporphyrin (SnPP, an HO-1 inhibitor) on the rat neonatal model of hyperbilirubinema. Serum bilirubin levels of rats treated by siRNA-4 and SnPP were significantly attenuated compared with the control group. However, siRNA-4 exhibited more inhibitory effect on indirect bilirubin than SnPP (Figure 10A).

Real-time PCR also showed that HO-1 level in the liver significantly decreased in the rats treated with siRNA-4 but not in those treated with SnPP (Figure 10B). These results reveal that SnPP possesses a dual role: it could up-regulate HO-1 expression but significantly inhibit HO-1 activity.

## Discussion

Bilirubin exerts a mixture of helpful and harmful effects. Positive effects include its role as an anti-oxidant and anti-inflammatory agent [8,9]. However, the abnormal increase of total serum bilirubin, particularly indirect bilirubin (unconjugated bilirubin) in the neonatal period can cause devastating neurological complications. Indirect bilirubin, a lipid-soluble molecule, is taken up by hepatic cells and transformed into glucuronic acid bilirubin (direct bilirubin) in the presence of UDP-glucuronyl transferase. Lipid-soluble indirect bilirubin can penetrate the cell membrane and the blood brain barrier, leading to neonatal kernicterus, whose central nervous system sequelae reflect both a predilection of bilirubin toxicity for neurons (rather than glial cells) and the regional topography of bilirubin-induced neuronal injury involving prominent basal ganglia, cochlear and oculomotor nuclei [10].

Currently, the treatments for neonatal hyperbilirubinnemia are traditional phototherapy, exchange transfusion and drug treatment [11-13]. Although these treatments are effective, they are limited to removing excessive bilirubin rather than preventing the production of bilirubin. Bilirubin is a product of complex heme metabolism that requires the participation of a series of key enzymes. HO is an inducible, rate-limiting enzyme in the production of bilirubin. ALA is a heme precursor which can rapidly and consistently cause elevation of the serum bilirubin levels. Concurrent with increasing bilirubin, hepatic heme oxygenase activity increases considerably due to the increased availability of ALA-derived heme [3]. Recently, HO-1 has become a novel therapeutic target for attempts to reduce bilirubin production. Sn-mesoporphyrin (SnMP) and SnPP, which are structural analogs of heme, competitively inhibit HO enzymatic activity and consequently decrease the synthesis of bilirubin [14]. Clinical trials have demonstrated that SnMP effectively prevents hyperbilirubinemia caused by premature birth and hemolysis [15,16]. However, the inhibitory mechanism of SnMP on hyperbilirubinemia *in vivo *is uncertain [17]. Therefore, the safety issue concerning the application of this compound in clinics remains to be fully studied.

In this study, we investigated whether siRNAs can effectively inhibit HO-1 expression that is up-regulated by hemin under hyperbilirubinemia conditions. Recent progress in siRNA technology provides a novel means to treat many diseases including hyperbilirubinemia. siRNA shows a superior specificity and stability compared with antisense RNA [18-20]. Since HO-1 is an inducible enzyme, targeted suppression by siRNA is a promising therapeutic approach to suppress HO-1 both *in vitro *and *in vivo*.

We first compared four pairs of siRNAs for their effects on inhibiting rHO-1 in BRL cells. We found that siRNA-4 was the most potent inhibitor of HO-1 expression and activity, and its inhibitory effect was both dose- and time-dependent. To further test if siRNA-4 could suppress HO-1 induced by endogenous heme *in vivo*, we established an animal model of neonatal hyperbilirubinemia according to the report of Drummond *et al *[3]. Seven to 9-day-old neonatal SD rats were treated with ALA intraperitoneally. These animals subsequently developed hyperbilirubinemia that mimics human hyperbilirubinemia. Injection of siRNA-4 significantly inhibited the rHO-1 activity in the liver and reduced the levels of serum bilirubin in the hyperbilirubinemia rats. SnPP, as a chemical inhibitor, could also reduce the serum bilirubin levels. However, the indirect bilirubin of siRNA-4 treated rats decreased more significantly. Taken together, our results suggest that siRNA may hold for the treatment of hyperbilirubinemia conditions in human neonates.

Although siRNA has been widely applied to therapeutic research on various diseases, there are still many difficulties that prevent its clinical application at the present time. One of the major challenges is the stability of siRNA *in vivo*. After entering the body, unmodified siRNA is quickly degraded by endogenous nuclease [21]. Various methods have been employed to stabilize the siRNA. For example, certain vectors are used to synthesize cationic polymer. Additionally, chemical modification of siRNA can improve siRNA stability and enhance its delivery efficacy as well as therapeutic specificity [22]. As demonstrated in our study, the therapeutic effect of methoxy-siRNA can last more than 48 hours after a single administration. Previous studies have shown that similar modification of siRNA can enhance the affinity of siRNA to target sequences, improve resistance to degradation by nuclease, and increase the interference of a target gene [23].

Another obstacle hindering siRNA treatment progress is that siRNA may not reach its therapeutic target or organ. Presently, this obstacle is overcome by local administration of siRNA. For instance, siRNA targeting vascular endothelial growth factor for age-related macular degeneration is delivered through retinal injection. In addition, siRNA can treat chronic neuropathic pain through intrathecal administration [24,25]. Systemic delivery of siRNA is used to target multiple organs [26]. For example, siRNA administrated intraperitoneally is mainly distributed in the liver, spleen and bone marrow [26]. It is well documented that HO-1 is primarily expressed in liver and spleen. Thus, we selected the intraperitoneal injection as the administration method for this study. We demonstrated that siRNA-4 was mainly distributed in the liver and spleen, and both serum bilirubin levels and HO-1 expression were significantly reduced in a dose- and time-dependent manner after intraperitoneal injection of siRNA. The maximal inhibitory effect was observed at 48 hours after single injection of siRNA. These findings are consistent with other published studies [27].

## Conclusion

Compared to the traditional phototherapy and exchange transfusion, the mechanism of siRNA treatment is different from the traditional methods in view of directly inhibiting the production of bilirubin. Our study provides a novel rationale to treat neonatal hyperbilirubinemia via further development of RNA interference techniques.

## Methods

### siRNA Design and Synthesis

Five pairs of siRNA were designed based on the rHO-1 mRNA sequence and purchased from Shanghai GenePharma Co., Ltd. siRNA-1, sense 5'-CGA GGU GGG AGG UAC UCA UTT-3', antisense 5'-AUG AGU ACC UCC CAC CUC GTG-3'. siRNA-2, sense 5'-GGG UGA CAG AAG AGG CUA ATT-3', antisense 5'-UUA GCC UCU UCU GUC ACC CTG-3'. siRNA-3, sense 5'-GGG AAU UUA UGC CAU GUA ATT-3', antisense 5'-UUA CAU GGC AUA AAU UCC CTT-3'. siRNA-4, sense 5'-CCG UGG CAG UGG GAA UUU ATT-3', antisense 5'-UAA AUU CCC ACU GCC ACG GTT-3'. NC-siRNA, sense 5'-UUC UCC GAA CGU GUC ACG UTT-3', antisense 5'-ACG UGA CAC GUU CGG AGA ATT-3'. 5' terminal FAM labeled NC-siRNA sense strand was used to determine siRNA transfection efficiency. All of the siRNAs were modified by methoxy for rat injection.

### Cell Culture and siRNA Delivery

BRL cells, a rat liver cell line obtained from the Shanghai Cell Bank of the Chinese Academy of Sciences, were used in the study. The cells were cultured in DMEM-High glucose media (Invitrogen) supplemented with 10% FBS (Hyclone Co.) at 37°C under 5% CO_2_. Transfection was carried out according to the manufacturer's protocol (Polyplus-transfection Inc.). Briefly, the cells were seeded in a 12-well plate and cultured for 24 hours to reach 30%–50% confluence. Each well was replaced with 1 ml fresh media prior to transfection. Five microliters of 2 μmol/L siRNAs were diluted with OPTI-MEM to 200 μl and were mixed with 5 μl INTERFERIN tranfection reagent (Polyplus-transfection Inc.). The mixture was incubated at room temperature for 10 minutes and was then added into each well. After slight shaking, the cells were incubated at 37°C for further experiments.

The transfection efficiency was determined using the NC-siRNA. Cells were transfected with FAM labeled NC-siRNA (50 nmol/L, final concentration). After 6-hour incubation, the cells were trypsinized and harvested by centrifugation. The siRNA transfection efficiency was observed under confocal microscopy and determined by flow cytometry.

### Optimization of the Dosage and Time of siRNA Delivery

Four different pairs of double-stranded siRNAs (10 nmol/L each) were transfected into BRL cells, respectively. Twenty-four hours later, total RNAs were isolated and rHO-1 expression was determined by RT-PCR using GAPDH as the internal control. Inhibitory effect of each siRNA on rHO-1 mRNA was determined and compared. The siRNA that exhibited the most potent inhibitory effect was selected and transfected to BRL cells at different doses (1, 10 and 50 nmol/L). Twenty-four hours later, the cells were harvested and rHO-1 expression was analyzed by RT-PCR to determine the optimal dosage of the siRNA.

The selected siRNA with the maximum inhibitory effect on HO-1 was transfected at the optimized dosage into BRL cells. The rHO-1 expression was analyzed at 15, 24 and 48 hours after transfection to determine the best time point for the inhibition.

The primer sequences used for RT-PCR analysis (synthesized by Invitrogen) are as follows: *HO-1 *Forward: 5'-CGG CCC TGG AAG AGG AGA TAG-3', Reverse: 5'-CGA TGC TCG GGA AGG TGA AAA-3'. *GAPDH *Forward: 5'-GTC GTG GAG TCT ACT GGC GTC TT-3', Reverse 5'-CAG TCT TCT GAG TGG CAG TGA TGG-3'. The PCR was carried out at the following condition: pre-denaturing at 94°C for 4 minutes, followed by 35 cycles of denaturing at 94°C for 30 seconds, annealing at 60°C for 30 seconds, and extension at 72°C for 30 seconds. For GAPDH, 28 cycles was used instead of 35. The final extension was 72°C for 10 minutes. The PCR products were checked by agarose gel and scanned by a gel documentation system (UVP). The results were analyzed by the quantitative software, Labworks 4.5.

### Modulation of rHO-1 Expression

BRL cells were treated with 1.5, 4.5 and 15 μmol/L hemin for 18, 24 and 36 hours, respectively, and were then collected by centrifugation at 6,000 rpm for 5 minutes. The cells was boiled with 20 μl 2×SDS loading buffer for 10 minutes and centrifuged at 10,000 rpm for 3 minutes to collect supernatants. Then the supernatants were separated by 12% SDS-polyacrylamide gel and transferred to PVDF membrane. HO-1 IgG (1:200, Sigma-Aldrich) followed by HRP-goat anti-rabbit antibody (1:10,000, Kangcheng Co.), and mouse anti-GAPDH antibody (1:10,000, Kangcheng Co.) followed by HRP-goat anti-mouse antibody (1:10,000, Kangcheng Co.) were added on the membrane respectively, for 1 hour. rHO-1 and GAPDH were visualized by EZ-ECL detection (Biond Co.), and analyzed by gray scale scanning quantitation.

BRL cells were transfected with the effective dose of siRNA for 24, 36 and 48 hours. At the end of transfection, 4.5 μmol/L hemin was added to induce the expression of HO-1 protein for 18 hours before harvesting. The protein levels were detected by Western Blot.

### The rHO-1 Activity Assay

Cells seeded in a 10-cm plate were transfected with the effective dose of siRNA-4 followed by treatment with 4.5 μmol/L hemin for 18 hours. The cells were lysed at 4°C, and the supernatant was collected by centrifuging at 14,000 rpm for 10 minutes. Ten grams of fresh liver tissue from SD rat (Shanghai SLAC Laboratory Animal Co.) were added into 20 ml of 0.1 mol/L potassium phosphate buffer (pH 7.4), homogenized, and centrifuged at 40,000 rpm for 1 hour at 4°C. The middle level aqueous phase containing biliverdin reductase was collected. The protein concentration was measured using BAC kit (Pierce, Rockford, IL) according to manufacturer's instruction. The enzyme-catalyzed system included 10 nmol/L hemin, 20 nmol/L β-nicotinamide adenine dinucleotide phosphate hydrogenase (β-NADPH, Sigma-Aldrich), 1 unit/μl Glucose-6-phosphate dehydrogenase (G-6-PD, Sigma-Aldrich), 1.17 mol/L Glucose-6-phosphate (G-6-P, Sigma-Aldrich), 25 mmol/L Magnesium Chloride (MgCl_2_), an aliquot of biliverdin reductase and cell supernatant. The reaction was carried out at 37°C for 1 hour and ceased by ice. The samples were scanned with a spectrophotometer (Shima-dzu, Tokyo, Japan) at absorbance from 464 to 530 nm. Bilirubin concentration was calculated based on the change of optical density from 530 to 464 nm, with an extinction coefficiency of 40 mmol^-1^cm^-1^. rHO-1 activity was expressed as nanomole of bilirubin per milligram of protein per hour (nmol bilirubin mg protein^-1 ^h^-1^).

### The Distribution of siRNA in Neonate Rat

The distribution of siRNA-4 was analyzed in neonate rat to confirm targeting effect of siRNA-4 *in vivo*. FAM labeled siRNA-4 was introduced into 7-day-old neonate SD rat with 5 OD/20 g bw by intraperitoneal injection. At 6, 24 and 48 hours after injection, the animal was anesthetized with aether and the liver, spleen, kidney, heart and lung were immediately removed and frozen in liquid nitrogen, then stored at -70°C. All tissues were embedded in Optimal Cuttin Temperature (OCT, SAKURA, USA) and sections (7 μm) were cut from the blocks, then immediately fixed in cold acetone for 30 minutes. Slides were stained with DAPI and washed in PBS for three times. The distribution of FAM-siRNA in each tissue was observed under confocal microscopy (Nikon A1-R, Japan).

### Establishment of Neonatal Hyperbilirubinemia Rat Model

A neonatal hyperbilirubinemia rat model was established according to a published protocol [3]. Seven to 9-day-old SD rats were treated intraperitoneally with ALA (Sigma-Aldrich) for 16, 20, and 24 hours, respectively at 50 μmol/100 g bw. These animals were breast-fed in a light-free condition. After the development of hyperbilirubinemia, the neonatal rats were anesthetized with ether prior to a blood draw. For each rat, 0.5 ml of blood was drawn from the right ventricle through the cardiac apex. The serum was separated and preserved under light-free conditions to determine the serum bilirubin levels (Beckman CX5 automatic biochemistry analyzer). The rats were sacrificed by cervical dislocation. The livers were harvested, preserved in liquid nitrogen, and immediately processed to assay rHO-1 level.

### Effects of siRNA and SnPP on Neonatal Hyperbilirubinemia Rats Models

To optimize the effective dosage of siRNA, seven-day-old SD rats were randomly divided into 4 groups. siRNA was injected into three groups of mice intraperitoneally at 0, 8 and 24 hours with 3, 5 and 10 OD/20 g bw, respectively. The control group was injected with 0.9% sodium chloride instead. ALA was injected to all the groups at 48 hours. At 72 hours, rat blood was taken to determinate the serum bilirubin levels and liver samples were separated to determinate the protein levels of rHO-1.

To optimize the time of siRNA treatment, seven-day-old SD rats were randomly divided into 4 groups. The siRNA group were treated intraperitoneally with 5 OD/20 g bw of siRNA on day 7, 8 and 9 after birth, respectively. The SnPP group were treated intraperitoneally with 75 μmol/kg bw of SnPP on day 7 and 8 after birth, respectively. Control group was injected with 0.9% sodium chloride or NC-siRNA instead on day 7, 8 and 9 after birth. Each group was injected with 50 μmol/100 g bw of ALA on day 9 after birth. The effect of siRNA on HO-1 was assessed with the comparison to SnPP. Twenty-four hours after ALA injection, the blood and liver samples were obtained to determinate the levels of serum bilirubin and rHO-1 expression.

All animal experiments in this study received prior approval from the Ethics Committee of Ruijin Hospital, Medical School, Shanghai Jiaotong University under experimental animal license SYXK (Shanghai 2008-0050).

### Mesurement of HO-1 mRNA in the Liver by Real-Time PCR

Rat liver samples were homogenized and total RNA was extracted by Trizol (Invitrogen Life Technologies). Real-time PCR was performed using an ABI Prism 7900HT (Applied Biosysterms, Foster City, CA) according to the manufacturer's instructions. Primers specific for *GAPDH*: forward: 5'-TGC ACC ACC AAC TGC TTA G-3' and reverse: 5'-GAT GCA GGG ATG ATG TTC-3', *HO-1*: forward: 5'-GCT AGC CTG GTT CAA GAT AC-3' and reverse: 5'-CAA CAG GAA ACT GAG TGT GA-3'. Primers were designed and synthesized by Invitrogen Corporation. Reaction conditions were 2 minutes at 50°C, 10 minutes at 95°C, followed by 40 cycles of 95°C for 15 seconds, 60°C for 1 minute and 50°C for 15 seconds, finally 60°C for 15 seconds and 95°C for 10 minutes. The expression of target genes was normalized to the expression of *GAPDH*.

### Statistical Analysis

Data were presented as the mean ± SD. Statistical comparisons were made by one-way ANOVA analysis in Figure 2, 3, 8, 9, 10. Student *t*-test was applied in Figure 5 and 7.

## List of abbreviations

ALA: δ-aminolevulinic acid; bw: body weight; DAPI: 4',6-diamidino-2-phenylindole; HO: heme oxygenase; NC: negative control; SD: Sprague-Dawley; siRNA: small interference RNA; SnMP: Sn-mesoporphyrin; SnPP: Sn-protoporphyrin.

## Authors' contributions

WJY and SW contributed equally to this work. WJY carried out the majority of the experiments, with assistance from JYX and SY. SW observed and analyzed the distribution of FAM-siRNA-4 and examined serum bilirubin levels of rats treated with siRNA and SnPP. LCE and ZWW performed quantitative PCR and some biochemistry assays. ZXH conducted statistical and pathological analyses. ZZL participated in the study design. XZW proposed and designed the project, and revised the manuscript. All authors discussed the results and approved the final manuscript.
